# Prescribed Rest Intervals in Home-Based Exercise Therapy and Rehabilitation: A Narrative Review and Conceptual Framework for Temporal Fidelity

**DOI:** 10.3390/healthcare14182911

**Published:** 2026-09-09

**Authors:** Nouf H. Alkhamees, Marilyn Gilyana, Sameer Badri Al-Mhanna, Alexios Batrakoulis

**Affiliations:** 1 Department of Rehabilitation Sciences, College of Health and Rehabilitation Sciences, Princess Nourah bint Abdulrahman University, P.O. Box 84428, Riyadh 11671, Saudi Arabia; 2 College of Sport Science, University of Kalba, Kalba P.O. Box 11115, Sharjah, United Arab Emirates; marilyn.gilyana@ukb.ac.ae; 3 Department of Higher Studies, Al-Qasim Green University, Babylon 51013, Iraq; sameerbadri9@gmail.com; 4 Department of Life Sciences, European University Cyprus, Nicosia 2404, Cyprus; 5 Department of Physical Education and Sport Science, Democritus University of Thrace, 69100 Komotini, Greece

**Keywords:** exercise therapy, rehabilitation, treatment adherence and compliance, telerehabilitation, rest intervals, home-based exercise, exercise adherence

## Abstract

Adherence to home-based exercise programs is central to rehabilitation, yet adherence to prescribed rest intervals remains largely unexplored. This narrative review synthesized relevant literature identified through targeted, purposive searches of PubMed, Scopus, Web of Science, and Google Scholar for publications from 2000 to 20 July 2026, supplemented by reference-list screening. Direct evidence is extremely limited: no study identified prospectively quantified adherence to prescribed rest intervals in a home-based rehabilitation setting. Accordingly, most interpretation is extrapolated from broader literature on general exercise adherence, resistance training, telerehabilitation, and digital monitoring. Rest duration can influence neuromuscular recovery, fatigue, and performance, providing a physiological rationale for studying recovery timing, but these effects do not establish the clinical consequences of rest-interval non-adherence. We distinguish participant adherence from intervention fidelity and propose temporal fidelity as a hypothesis-generating framework describing concordance between the intended and implemented timing structure of exercise. Explicit prescriptions, patient education, simplified timing cues, supervised familiarization, and digital monitoring are presented as candidate strategies rather than established rest-specific interventions. Prospective studies should objectively measure prescribed and actual recovery periods and determine whether temporal fidelity predicts or improves rehabilitation outcomes.

## 1. Introduction

Home-based exercise programs are widely implemented across rehabilitation and preventive health settings due to their accessibility, scalability, and cost-effectiveness. They play a central role in supporting long-term self-management, particularly in areas with limited access to supervised care. Despite these advantages, adherence remains a persistent, multifactorial challenge, with considerable variability in how individuals engage with and perform prescribed exercises [1,2]. Adherence is typically defined as the extent to which an individual’s behavior corresponds with agreed recommendations, commonly operationalized through dimensions such as frequency, intensity, and duration [3].

In this review, adherence and fidelity are treated as related but distinct constructs. Adherence refers to the participant’s behavior in relation to an agreed prescription, whereas intervention fidelity refers more broadly to the degree to which an intervention is delivered and implemented as intended. In a home-based program, rest-interval adherence is therefore the observable participant behavior (for example, whether a prescribed 90-s recovery period is followed), while temporal fidelity is the proposed broader construct describing concordance between the intended and implemented timing structure. Participant adherence to prescribed recovery timing may contribute to temporal fidelity, but the terms are not used interchangeably.

Rest intervals between sets or exercises are a distinct component of exercise prescription. Evidence from resistance-training research indicates that rest duration can influence acute performance, fatigue accumulation, and the ability to sustain force production across repeated efforts [4,5]. However, evidence that rest duration affects exercise physiology does not demonstrate that patients fail to follow prescribed rest intervals in home-based rehabilitation, nor does it establish the clinical consequences of such non-adherence. Direct evidence addressing this behavioral question remains scarce.

Contemporary behavior-change and implementation frameworks emphasize fidelity to intervention components [6,7]. Nevertheless, the clinical relevance of rest timing is likely to vary according to diagnosis, exercise modality, intensity, therapeutic goal, age, comorbidity, symptom response, and whether recovery is fixed or self-regulated. Rest intervals should therefore not be assumed to have equal importance across all rehabilitation programs.

On this basis, we propose ‘temporal fidelity’ as a hypothesis-generating timing-specific framework encompassing rest duration, pacing, and recovery structure. The term is not presented as an established construct in rehabilitation science. Rather, it provides a testable way to describe whether the intended temporal component of an exercise prescription is implemented as planned while preserving the conceptual distinction between participant adherence and broader intervention fidelity.

Broader home-based rehabilitation literature documents substantial challenges with overall exercise adherence and self-management [8,9,10,11], while specific prescription components are less often measured objectively [12]. Digital-health, wearable-monitoring, and artificial-intelligence approaches have been explored for general exercise support and remote rehabilitation [13,14,15,16]; however, their usefulness for rest-interval adherence remains unestablished and is considered later as a potential strategy rather than an assumed solution.

Against this background, the central question is: What evidence exists regarding adherence to prescribed rest intervals in home-based rehabilitation, how has this adherence been measured, and what is known about its behavioral and clinical implications? This review therefore aims to (1) distinguish direct evidence on rest-interval behavior from indirect rehabilitation and contextual exercise-science evidence, (2) examine potential behavioral and clinical implications without conflating physiological effects with adherence, and (3) propose a conceptual framework and priorities for future research. An additional purpose is to clarify what should be measured when this question is tested prospectively. Distinguishing the prescribed timing target from the recovery period actually performed allows adherence to be quantified as a behavioral variable rather than inferred from attendance or session completion. This distinction may also help researchers identify whether timing deviations are systematic, situational, or simply a consequence of an inadequately specified exercise prescription.

## 2. Narrative Review Approach

This narrative review was undertaken to develop a critical, conceptually focused synthesis of literature relevant to prescribed rest intervals in home-based rehabilitation, consistent with principles for narrative literature reviews [17]. Targeted searches were conducted in PubMed, Scopus, Web of Science, and Google Scholar for publications from 2000 onward and were last updated on 20 July 2026. Search concepts included combinations of ‘home exercise,’ ‘rehabilitation,’ ‘exercise adherence,’ ‘rest intervals,’ ‘exercise fidelity,’ ‘telerehabilitation,’ and ‘self-management,’ and reference lists of key reviews and highly relevant articles were examined. Because source identification was purposive rather than exhaustive, the review was not designed as a reproducible systematic search: database-specific Boolean strings, record-level screening counts, and a PRISMA flow diagram were therefore not used. The final narrative synthesis cites 102 sources; this number reflects references used for evidence synthesis and contextual framing and should not be interpreted as a systematic-review count of formally included studies.

Home-based rehabilitation was considered therapeutic exercise performed primarily outside a clinical facility without continuous in-person supervision. Clinical rehabilitation populations were prioritized. Studies involving healthy or general fitness participants were considered only when they provided contextual exercise-science evidence directly relevant to rest physiology, rest behavior, or exercise fidelity. Resistance, aerobic, interval/circuit, and multimodal exercise were considered where relevant. Primary empirical studies, systematic or scoping reviews, guidelines, conceptual papers, and digital-health studies were eligible for contextual inclusion; these source types were not treated as having equivalent evidentiary strength.

For synthesis, evidence was classified a priori by directness: (1) direct rest-interval evidence, in which actual rest or recovery behavior was prescribed or measured; (2) indirect rehabilitation evidence, addressing overall adherence, attendance, exercise frequency, training volume, or intervention fidelity in home-based or rehabilitation settings; and (3) contextual exercise-science evidence, including physiological effects of rest duration, supervised versus unsupervised training, and digital monitoring outside the target setting. No study identified met the stricter criterion of both prescribing rest intervals and prospectively measuring adherence to those prescribed intervals in a home-based rehabilitation setting. Study characteristics considered during synthesis included population, clinical condition, exercise setting and modality, adherence measure, whether rest was prescribed or measured, and principal findings. Evidence was grouped thematically and qualitatively weighted according to directness, study design, and source type; directly relevant primary empirical studies and systematic reviews were given greater interpretive weight than narrative, conceptual, guideline, conference, or preprint sources. No formal independent duplicate screening or risk-of-bias assessment was conducted, consistent with the narrative-review design. Heterogeneity and methodological limitations were therefore incorporated into the interpretation rather than converted into a formal certainty-of-evidence rating. This hierarchy was used to avoid assigning the same interpretive weight to evidence that addressed different questions. In particular, physiological studies were used to explain why recovery timing may matter, whereas rehabilitation-adherence studies were used to identify plausible behavioral influences. Neither evidence stream was treated as a substitute for direct prospective measurement of prescribed versus performed rest intervals in the target setting. A narrative evidence map summarizing the contributing populations/domains, typical evidence sources, and their level of directness is presented in Table 1.

## 3. Results: Thematic Evidence Synthesis

Evidence is organized below as a thematic narrative synthesis. The primary emphasis is on evidentiary directness: findings that measure rest behavior are separated from rehabilitation studies of general adherence and from contextual exercise-science evidence.

### 3.1. Rest Intervals: Physiological Rationale and Supporting Evidence

Rest intervals are a key variable in exercise prescription and can influence metabolic recovery, neuromuscular performance, and the capacity to sustain force and technical execution across repeated efforts. Evidence from exercise and resistance-training studies shows that rest duration affects phosphocreatine resynthesis, fatigue accumulation, and performance [67,68]. These findings establish the physiological relevance of recovery timing, but they do not directly demonstrate that patients adhere to prescribed rest intervals in home-based rehabilitation or that deviations alter clinical outcomes.

There is also evidence that demanding exercise prescriptions can affect overall adherence, highlighting the need to balance workload and recovery [69,70]. Experimental evidence suggests that insufficient recovery between efforts may increase perceived fatigue and alter performance or motor control. However, extrapolating these acute physiological effects to clinical consequences of rest-interval non-adherence in home-based rehabilitation should be done cautiously because direct prospective evidence is lacking.

Rest duration may be particularly relevant in rehabilitation populations with reduced physiological reserve or conditions requiring careful control of exercise stress [25,26]. In cardiac rehabilitation, for example, recovery periods can be used to manage hemodynamic responses and cardiovascular stability [27]. Nevertheless, the literature primarily addresses how rest should be prescribed rather than whether patients actually follow prescribed recovery times at home. Thus, the physiological rationale for rest intervals is stronger than the evidence concerning adherence to them [28,29,30,31,32,33].

### 3.2. Physiological Rationale and Context Dependence

One important methodological challenge is the lack of standardized tools to objectively measure adherence to prescribed rest intervals in home-based settings. Most studies rely on self-reported exercise diaries, which are vulnerable to recall bias and overestimation of adherence. Emerging wearable technologies may offer more objective approaches; however, their validity and feasibility in rehabilitation populations remain insufficiently established. Measurement precision is especially important because a recorded exercise session can appear fully completed even when its temporal structure differs substantially from the prescription. A useful assessment approach would therefore record both the target recovery period and the observed recovery period for each relevant exercise bout. Reporting the magnitude and direction of this deviation would permit temporal fidelity to be examined independently from attendance, repetition completion, and total exercise volume.

Although specific research on patient adherence to rest intervals is notably sparse, broader evidence on home-based exercise programs provides a framework for understanding adherence behaviors. Overall adherence rates for unsupervised regimens are typically suboptimal, ranging from 50% to 70% (e.g., ~67% average adherence; non-compliance 30–50%) in musculoskeletal cohorts [8,19]. These data concern overall exercise adherence rather than compliance with prescribed rest intervals and are therefore interpreted here as indirect evidence.

Similarly, evidence linking higher adherence with better pain and physical-function outcomes, and studies showing improved adherence with structured or digital rehabilitation, primarily assess broad adherence dimensions such as attendance, frequency, duration, or completion [20,21]. Existing adherence measures also largely capture these broader dimensions, while finer prescription components such as rest intervals are rarely monitored [22,23,24].

Direct evidence on rest-interval behavior remains extremely limited. No study identified in this narrative synthesis both prescribed a rest interval within a home-based rehabilitation program and prospectively measured patient adherence to that prescribed interval. Silva et al. [18] directly profiled self-selected rest intervals among general resistance-training participants and reported that 71.6% attempted to manage rest periods, while approximately 95% of those tracking rest selected intervals of 60 s or less. This study was conducted in a general training population rather than in home-based rehabilitation and did not evaluate adherence to a therapeutic rest prescription. Other cited evidence addresses exercise fidelity, supervised versus unsupervised training, or broader adherence and therefore only indirectly informs prescribed rest-interval adherence [34,35,36,71,72,73].

Accordingly, the clinical and behavioral implications discussed below should be interpreted according to this evidentiary distinction: findings directly measuring rest-interval behavior are identified as such, whereas evidence derived from general exercise adherence is used only to support conceptual extrapolation to temporal fidelity.

For clarity, the adherence outcomes discussed across the cited literature are not interchangeable. Session attendance or completion, exercise frequency, total training volume, adherence to work-to-rest ratios, and actual inter-set rest duration represent distinct constructs. Only measures of prescribed timing or actual recovery duration directly inform temporal fidelity when a timing target is explicitly specified.

Rest prescription itself is also context-dependent. Longer recovery is not universally preferable: shorter rest may be intentionally prescribed in circuit or interval training, whereas longer recovery may be selected to preserve force production, manage symptoms, or accommodate reduced physiological reserve [25,26,27,67,68]. In some low-risk or symptom-guided programs, self-regulated recovery may be clinically appropriate. Temporal fidelity should therefore refer to fidelity to the intended, individualized timing strategy rather than adherence to a universally fixed or longer rest interval.

### 3.3. Potential Determinants of Rest-Interval Adherence: Indirect Evidence

The evidence summarized in this subsection primarily concerns overall home-based exercise adherence rather than prescribed rest intervals specifically and is therefore used only as an extrapolative framework for potential determinants of rest-interval adherence. Studies have identified patient understanding, low self-efficacy, poor social support, and perceived barriers as important correlates of general exercise or physiotherapy adherence [1,73]. If similar relationships apply to timing behavior, they could influence temporal fidelity, but this has not been directly demonstrated.

Program simplicity, intrinsic motivation, perceived efficacy, and emotional state have also been associated with broader adherence or engagement [19,74,75,76,77,78,79]. These studies did not quantify compliance with prescribed recovery times; consequently, their relevance to rest-interval adherence is plausible but unconfirmed.

Economic circumstances, resource availability, perceived ability to succeed, and communication with healthcare providers likewise influence general rehabilitation adherence [80,81]. These determinants may affect patients’ capacity to self-manage prescribed timing at home, but this proposed relationship has not been directly tested. These potential determinants may also operate differently across the course of rehabilitation. Early in a program, unfamiliarity with instructions and uncertainty about symptom responses may be particularly influential, whereas later deviations may reflect convenience, habit formation, perceived lack of benefit, or deliberate self-adjustment. Longitudinal measurement would therefore be preferable to a single summary estimate of adherence because it could identify when and under what circumstances timing behavior changes.

#### 3.3.1. Behavioral Factors

The behavioral evidence cited here evaluates overall exercise participation, intensity monitoring, program complexity, routine formation, or engagement rather than prescribed-rest adherence [10,35,37,82,83,84]. Prior exercise experience, simpler prescriptions, self-monitoring, and established routines may plausibly influence rest-timing behavior, but their association with prescribed recovery periods remains theoretical.

#### 3.3.2. Psychological Factors

The psychological literature cited here evaluates overall rehabilitation adherence, engagement, or retention rather than prescribed-rest adherence [8,10,49,82,83,85]. Self-efficacy, perceived control, enjoyment, and emotional state are therefore presented as plausible influences on patient rest-timing behavior, not as established determinants of temporal fidelity. Similarly, interventions targeting motivation and psychological barriers have been studied in relation to general exercise adherence and fatigue management [40,82], not rest-interval adherence specifically.

#### 3.3.3. Environmental Factors

The environmental evidence likewise concerns general home-exercise adherence or participation rather than direct measurement of prescribed-rest compliance [50,82,83,84]. Social support, access to equipment, work schedules, socioeconomic circumstances, and professional guidance may plausibly influence how patients self-manage recovery timing at home; this extrapolation has not been directly evaluated.

### 3.4. Potential Clinical Relevance and Evidence Boundaries

Exercise-physiology evidence provides a plausible mechanism by which deviations from an intended recovery period could alter fatigue, force production, or movement kinematics [5,86]. However, no study identified in this narrative synthesis directly tested whether measured non-adherence to prescribed rest intervals in home-based rehabilitation predicts poorer clinical outcomes, movement quality, overuse pathology, or injury. These consequences should therefore be regarded as hypotheses supported by mechanistic and contextual evidence rather than established effects of temporal non-adherence. Findings in populations such as individuals with chronic low back pain, in whom fatigue and recovery can influence motor-control strategies [87,88], provide mechanistic context but do not demonstrate that rest-interval non-adherence causes secondary tissue strain or poorer rehabilitation outcomes.

Broader home-based rehabilitation literature identifies several factors that may support adherence, including supervised familiarization, self-monitoring, manageable participant burden, and ongoing professional contact [38,39,40,89]. These studies primarily evaluate general adherence, feasibility, or exercise performance rather than adherence to prescribed rest intervals specifically. They therefore provide indirect support for teaching patients how to self-manage timing parameters, but the effectiveness of such approaches for improving temporal fidelity requires direct evaluation.

**Figure 1:**
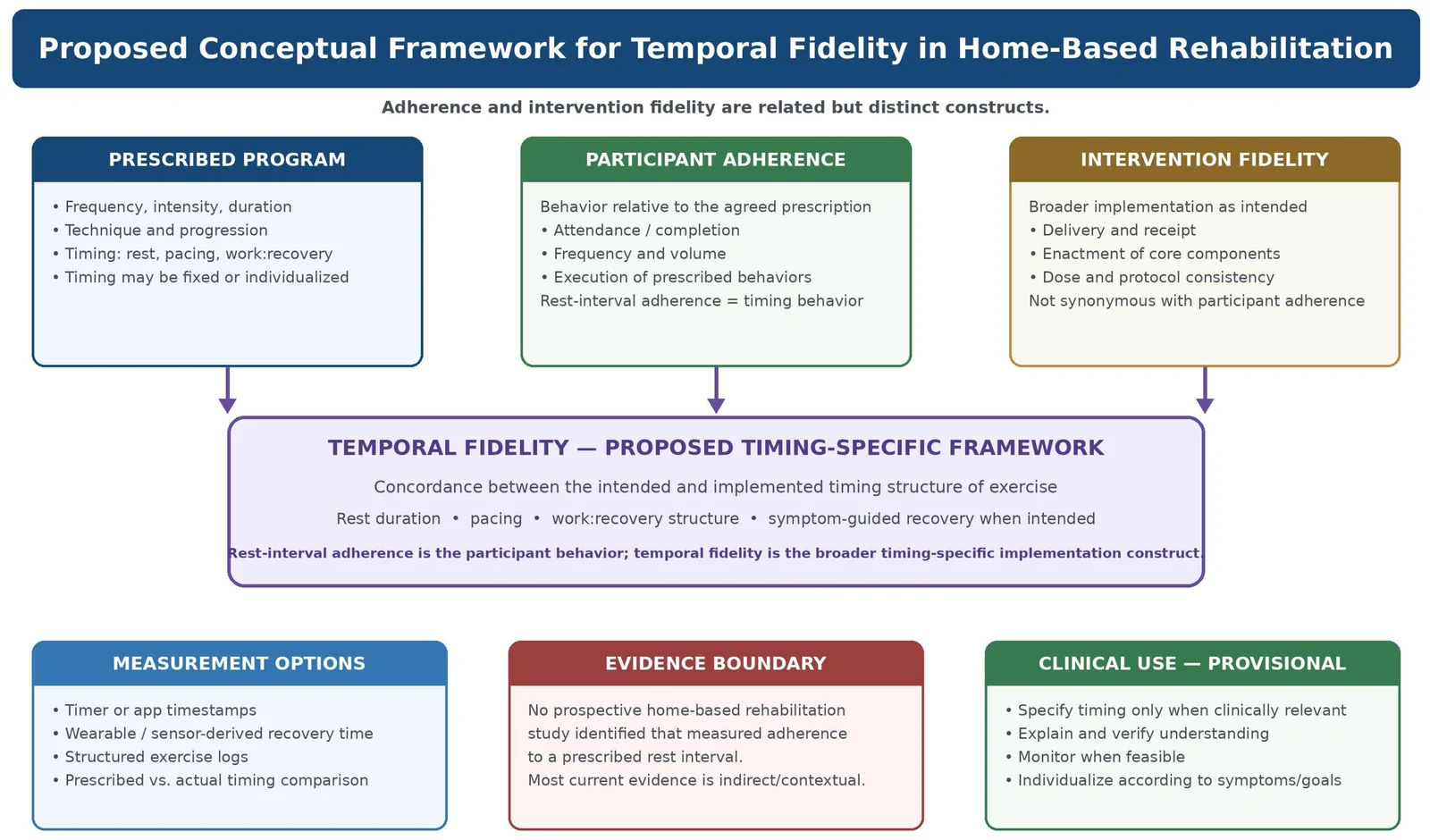
Proposed conceptual framework relating participant adherence, intervention fidelity, and temporal fidelity in home-based exercise rehabilitation. The framework distinguishes established evidence from hypotheses and provisional clinical strategies.

High-intensity exercise can reduce the time burden of training but may also be more difficult to sustain without supervision [51,52]. Supervised studies in middle-aged and older adults with T2D report high adherence to structured interval exercise [53], whereas transitions to home-based resistance training can be accompanied by reductions in exercise frequency and total training volume [54]. These findings concern overall training adherence and do not establish compliance with prescribed recovery periods in unsupervised settings. Figure 1 therefore presents a conceptual summary of why rest-interval adherence may warrant investigation rather than evidence of established clinical effects.

### 3.5. Candidate Strategies to Support Rest-Interval Adherence and Temporal Fidelity

Because direct evidence on interventions specifically targeting rest-interval adherence remains limited, the following strategies are primarily extrapolated from the broader home-based rehabilitation adherence literature. They may provide a practical framework for supporting temporal fidelity but require direct evaluation. Importantly, the goal of these candidate strategies should not be rigid compliance with a universal recovery duration. Their purpose would be to support accurate implementation of the timing strategy selected for a particular patient and therapeutic objective, including fixed, ranged, or symptom-guided recovery. This patient-centered interpretation preserves clinical flexibility while making the intended temporal component sufficiently explicit to be evaluated.

#### 3.5.1. Program Simplification and Patient Education

Reducing cognitive demand through clear, time-based instructions, individualized progression, and simplified routines may facilitate self-management [90,91,92]. Patient education should explain the purpose of prescribed recovery and provide simple timing cues; although psychoeducational approaches can improve self-efficacy and general physiotherapy adherence [1,61,62,73], their effects on rest-interval adherence specifically have not been established.

#### 3.5.2. Digital Monitoring and Structured Feedback

Smartphone apps, wearable sensors, digital logbooks, and real-time monitoring can replace subjective recall with structured feedback and may improve engagement in remote settings [58,59,60]. Wearable systems can also provide heart-rate and work-rest timing feedback [35,46], while telerehabilitation offers remote supervision and accountability [41,42,43,44,45,78]. However, evidence that these technologies specifically improve adherence to prescribed rest intervals remains limited.

#### 3.5.3. Behavioral, Social, and Supervised Support

Regular follow-up through counseling or digital messaging, simple monitoring tools or pictorial guides for patients less familiar with technology, and supervised familiarization may support adherence and self-management [40,47,48]. Family or caregiver support, culturally appropriate program adaptation, motivational interviewing, goal setting, and ongoing personalized feedback may further strengthen long-term engagement [47,93,94]. These approaches are supported primarily by general exercise-adherence evidence and should therefore be viewed as plausible strategies for temporal fidelity rather than established rest-interval-specific interventions.

## 4. Discussion

The main contribution of this review is not to establish that rest-interval adherence improves rehabilitation outcomes, but to clarify the current evidence boundary and define a testable research agenda. Three conclusions emerge from the synthesis: no prospective home-based rehabilitation study was identified that directly measured adherence to a prescribed rest interval; rest-interval adherence should be distinguished from general exercise adherence and from broader intervention fidelity; and temporal fidelity provides a hypothesis-generating framework for comparing intended with implemented exercise timing. Physiological evidence supports the plausibility of recovery timing as a component of exercise dose, but the clinical consequences of temporal non-adherence remain unconfirmed.

### 4.1. Conceptual Contribution and Generalizability Across Rehabilitation Populations

The broad scope of this review is intentional because direct evidence is too sparse to support a population-specific synthesis. Nevertheless, the contributing domains should not be treated as interchangeable. Musculoskeletal literature primarily informs general home-exercise adherence and measurement; cardiac rehabilitation contributes substantial contextual evidence on physiological monitoring, recovery, and telerehabilitation; obesity and type 2 diabetes studies inform structured interval/circuit exercise and transitions from supervised to home-based training; and general resistance-training research provides the limited direct evidence on rest behavior. The comparatively prominent cardiac literature reflects the availability of monitoring and remote-rehabilitation studies rather than stronger evidence for rest-interval adherence. Because diagnoses, exercise intensity, therapeutic goals, symptoms, and monitoring requirements differ, findings from one domain cannot be assumed to generalize to another.

Accordingly, temporal fidelity is proposed as a conceptual contribution that makes the evidence gap measurable: future studies can specify a timing target, quantify the difference between prescribed and actual recovery, and test whether that deviation is associated with symptoms, exercise quality, function, safety, or clinical outcomes. This moves the field from a general assertion that ‘rest matters’ toward a falsifiable question about whether adherence to timing prescriptions matters in particular rehabilitation contexts. This framework may also improve interpretation of intervention outcomes. If two participants complete the same number of sessions and repetitions but consistently use different recovery periods, they may receive meaningfully different patterns of exercise stress despite appearing equally adherent by conventional metrics. Incorporating temporal measures could therefore help explain otherwise unexplained variability in tolerance, progression, and response, while still requiring prospective evidence before any causal interpretation is made.

### 4.2. Practical Implications and Provisional Clinical Considerations

Given the limited direct evidence, the practical implications of temporal fidelity should be considered provisional. Clinicians may consider making recovery instructions explicit when timing is clinically relevant, confirming patient understanding, and allowing individualized or symptom-guided recovery where appropriate. These suggestions are extrapolated mainly from broader evidence that structured prescriptions, self-monitoring, professional support, and digital feedback can improve general exercise adherence [40,63,64,65,95,96,97,98]; they are not established rest-interval-specific interventions.

Similarly, mobile-health platforms, wearable devices, remote supervision, and adaptable video-based programs may provide feasible tools for monitoring or cueing recovery periods [66,99,100]. However, current studies largely examine general participation, physical activity, or program adherence. Their effectiveness for improving adherence to prescribed rest intervals, and whether such improvement translates into better rehabilitation outcomes, remains to be established. These provisional clinical considerations are summarized in Table 2.

These actions should be viewed as prudent implementation options rather than evidence-proven rest-specific practices. Their value lies in making recovery timing explicit and measurable when clinicians judge it to be relevant, thereby enabling future evaluation of temporal fidelity rather than assuming that a fixed rest interval is necessary across all rehabilitation programs.

### 4.3. Limitations and Future Research

This review has several important limitations. Source identification was purposive rather than exhaustive, independent duplicate screening was not performed, and no formal risk-of-bias or certainty-of-evidence assessment was undertaken. The 102 cited sources span heterogeneous study designs, populations, exercise modalities, and evidentiary strength, and most do not directly assess prescribed rest-interval adherence. The synthesis therefore depends substantially on indirect rehabilitation and contextual exercise-science evidence. Cardiac rehabilitation is disproportionately represented among technology and monitoring sources, which limits generalization to musculoskeletal, metabolic, neurological, and other rehabilitation populations. These limitations preclude firm conclusions about the prevalence, determinants, or clinical effects of rest-timing deviations.

Future research should prospectively prescribe recovery targets, objectively record actual rest duration, predefine temporal-fidelity metrics, and test whether deviations predict fatigue, symptoms, movement quality, safety, functional recovery, or other patient-centered outcomes. Comparative studies should also determine when fixed, individualized, or symptom-guided recovery is most appropriate across diagnoses and exercise modalities, and whether monitoring or feedback strategies improve timing behavior sufficiently to affect clinical outcomes. Future studies should additionally report the feasibility and burden of temporal monitoring, because a measurement strategy that substantially disrupts home exercise may itself alter behavior. Repeated assessments across the intervention period would help determine whether temporal fidelity is stable, improves with familiarization, or deteriorates as supervision decreases. Such designs could also distinguish intentional clinician-approved self-regulation from unplanned deviation, an important distinction for clinically meaningful interpretation.

## 5. Conclusions

Adherence to prescribed rest intervals is a plausible but unvalidated component of home-based rehabilitation. No study identified in this narrative synthesis prospectively measured adherence to a prescribed rest interval in a home-based rehabilitation setting; therefore, temporal fidelity should be regarded as a hypothesis-generating framework rather than an established clinical construct. When recovery timing is judged to be clinically relevant, clinicians may provisionally specify the intended timing strategy, explain its rationale, confirm patient understanding, monitor actual recovery where feasible, and individualize rest according to diagnosis, symptoms, intensity, and therapeutic goals. These actions are not yet evidence-proven rest-specific interventions. Prospective studies are required to determine whether objective monitoring and improvement of temporal fidelity produce measurable rehabilitation benefits. Accordingly, the immediate research priority is not to prescribe longer or more rigid rest periods, but to determine whether patients implement the recovery strategy that was actually intended and whether deviations have measurable consequences. Establishing this evidence would clarify whether temporal fidelity should become a routinely monitored component of home-based rehabilitation.

## Figures and Tables

**Table 1:** Narrative evidence map by population/domain and level of directness.

Population/Domain	Typical Evidence Used	Rest-Interval Adherence Measured?	Interpretive Role
General resistance-training participants	Rest-behavior profiling [18]	Actual/self-selected rest behavior measured; no therapeutic rest prescription	Contextual rest-behavior evidence only; not home-based rehabilitation
Musculoskeletal/home rehabilitation	Adherence reviews and observational studies [8,19,20,21,22,23,24]	No direct prospective measurement of adherence to prescribed rest	Indirect evidence on overall adherence, measurement, pain, and function
Cardiac/cardiovascular rehabilitation	Exercise prescription, monitoring, and telerehabilitation literature [25,26,27,28,29,30,31,32,33,34,35,36,37,38,39,40,41,42,43,44,45,46,47,48]	Recovery may be prescribed or monitored physiologically, but prescribed-rest adherence at home was not isolated	Important physiological and monitoring context; generalizability to other populations is limited
Metabolic/obesity/type 2 diabetes	Interval/circuit and supervised-to-home exercise studies [49,50,51,52,53,54,55,56,57]	Work–recovery structures may be prescribed, but rest adherence was not isolated	Indirect evidence on adherence, exercise completion, and training volume
Digital/telerehabilitation	Apps, wearables, remote monitoring, and feedback studies [13,14,15,16,21,41,42,43,44,45,46,58,59,60,61,62,63,64,65,66]	Mostly general engagement/adherence rather than prescribed-rest adherence	Context for candidate measurement and support strategies

**Table 2:** Provisional clinical considerations when recovery timing is considered clinically relevant.

Action	Practical Example	Evidence Status
Specify	State a target range or a symptom-guided recovery rule only when timing is clinically relevant.	Provisional; not validated as a rest-specific intervention
Explain	Explain why the timing strategy is used and how it relates to the exercise goal.	Extrapolated from patient-education/adherence evidence
Verify	Use supervised familiarization or teach-back to confirm understanding.	Extrapolated from broader rehabilitation practice
Monitor	Where feasible, compare prescribed and actual recovery using a timer, log, app, or wearable.	Measurement strategy requiring validation
Individualize	Adjust recovery according to diagnosis, symptoms, intensity, goals, and tolerance rather than enforcing one universal interval.	Consistent with context-dependent exercise prescription

## Data Availability

No new data were created or analyzed in this study. Data sharing is not applicable to this article.
